# Radical Scavenging and Anti-Inflammatory Activities of Representative Anthocyanin Groupings from Pigment-Rich Fruits and Vegetables

**DOI:** 10.3390/ijms19010169

**Published:** 2018-01-06

**Authors:** Federica Blando, Nadia Calabriso, Helge Berland, Gabriele Maiorano, Carmela Gerardi, Maria Annunziata Carluccio, Øyvind M. Andersen

**Affiliations:** 1 Institute of Sciences of Food Production (ISPA), National Research Council (CNR), Via Prov.le Lecce-Monteroni, 73100 Lecce, Italy; gabriele.maiorano@gmail.com (G.M.); carmela.gerardi@ispa.cnr.it (C.G.); 2 Institute of Clinical Physiology (IFC), National Research Council (CNR), Via Prov.le Lecce-Monteroni, 73100 Lecce, Italy; nadia.calabriso@ifc.cnr.it (N.C.); maria@ifc.cnr.it (M.A.C.); 3 Department of Chemistry, University of Bergen, Allegt 41, 5007 Bergen, Norway; Helge.Berland@kj.uib.no (H.B.); oyvind.andersen@kj.uib.no (Ø.M.A.)

**Keywords:** non-acylated anthocyanins, anthocyanins with aromatic acylation, structure–activity relationship (SAR), mahaleb cherry, blackcurrant, black carrot, “Sun Black” tomato, VCAM-1, ICAM-1, endothelial adhesion molecules.

## Abstract

Anthocyanins, the naturally occurring pigments responsible for most red to blue colours of flowers, fruits and vegetables, have also attracted interest because of their potential health effects. With the aim of contributing to major insights into their structure–activity relationship (SAR), we have evaluated the radical scavenging and biological activities of selected purified anthocyanin samples (PASs) from various anthocyanin-rich plant materials: two fruits (mahaleb cherry and blackcurrant) and two vegetables (black carrot and “Sun Black” tomato), differing in anthocyanin content (ranging from 4.9 to 38.5 mg/g DW) and molecular structure of the predominant anthocyanins. PASs from the abovementioned plant materials have been evaluated for their antioxidant capacity using Trolox Equivalent Antioxidant Capacity (TEAC) and Oxygen Radical Absorbance Capacity (ORAC) assays. In human endothelial cells, we analysed the anti-inflammatory activity of different PASs by measuring their effects on the expression of endothelial adhesion molecules VCAM-1 and ICAM-1. We demonstrated that all the different PASs showed biological activity. They exhibited antioxidant capacity of different magnitude, higher for samples containing non-acylated anthocyanins (typical for fruits) compared to samples containing more complex anthocyanins acylated with cinnamic acid derivatives (typical for vegetables), even though this order was slightly reversed when ORAC assay values were expressed on a molar basis. Concordantly, PASs containing non-acylated anthocyanins reduced the expression of endothelial inflammatory antigens more than samples with aromatic acylated anthocyanins, suggesting the potential beneficial effect of structurally diverse anthocyanins in cardiovascular protection.

## 1. Introduction

Anthocyanins are naturally occurring pigments responsible for the red to dark blue colour (in some cases perceived as black by the human eye) of most flowers, fruits and vegetables, and constitute a sub-class of flavonoids within the broad class of polyphenols. They are characterised by having, under acidic conditions, a common 2-phenylbenzopyrylium (flavylium) cationic aglycone with various oxygen functions (anthocyanidin), which may occur on other equilibrium forms when the pH in their surroundings changes. Twenty anthocyanidins having a C15 skeleton without skeleton extension are known to occur in plants; however, in nearly all fruits and vegetables only six of them are present: pelargonidin, cyanidin, delphinidin, peonidin, petunidin and malvidin. Anthocyanins are present in almost all plants as anthocyanidin glycosides. The sugar moieties (most often mono-, di- or tri-glycosides) can be further acylated with aliphatic and aromatic acids. More than 700 natural anthocyanins have been identified [1,2], revealing the plasticity of their biosynthetic pathway.

Over the last few decades, a huge amount of research has been focused on the possible health effects of anthocyanins relative to the consumption of fruit and vegetables containing these bioactive phytochemicals [3,4,5,6]. Many of these health activities have been attributed to the intense antiradical and antioxidant activity of anthocyanin. The relationship between anthocyanins’ chemical structure and their corresponding chemical and biological activity (structure–activity relationship, SAR) is a challenging area of research that has been tackled in several studies [7,8,9,10,11,12,13]. In these papers, the chemical features of anthocyanins (type of aglycone, type of glycosylation, non-acylated anthocyanins, acylated anthocyanins with aliphatic acids or with hydroxycinnamic acids) have been put in the context of biological activity. Here are some examples: The radical scavenging activity of anthocyanins has been strongly related to their chemical structure, including the substituents on the flavylium cation [14]. The type, position and number of hydroxyl/methoxyl groups, as electron-donating structures, have been considered pivotal features in relation to the antioxidant activity of anthocyanidins; the presence of 3′,4′-ortho-dihydroxyl groups in the flavylium B-ring [7,14] and a 3-hydroxyl in the C-ring [12] seem to represent important structural elements for anthocyanins in suppressing oxidative stress. Acylation of a sugar moiety will on the one hand increase the in vitro and in vivo chemical stability of the anthocyanin; however, on the other hand, a change in the ring orientation of the molecule causes hydrogen atom transfer from hydroxyl groups to unpaired electrons to be difficult, making acylated anthocyanins less potent antioxidants that non-acylated ones [13].

Data from epidemiological studies have shown an inverse relationship between anthocyanin intake and cardiovascular risk prevention [15] and mortality [16]. Several clinical and experimental studies with anthocyanins or anthocyanin-rich foods have demonstrated an improvement in vascular function [17,18] and a decrease in atherosclerotic plaque development [19,20,21]. The initial steps of atherosclerotic process consist of the recruitment and adhesion of circulating monocytes to endothelial cells and subsequent trans-endothelial migration into the intima of the vascular wall [22]. The adhesion of monocytes to the endothelium involves the concerted expression on the surface of the activated endothelium of adhesion molecules, such as vascular cell adhesion molecule-1 (VCAM-1) and intercellular adhesion molecule-1 (ICAM-1) [23], two pivotal vascular inflammatory antigens; therefore, their inhibition by natural compounds could counteract the atherosclerotic process and related *sequelae* of cardiovascular injuries. Recent studies have also documented the ability of anthocyanins to abrogate the adhesion of human monocytes to inflamed endothelial cells [24,25,26]; however, the relationship between different anthocyanin groupings and biological activities remains elusive.

With the aim of contributing major insights into the challenging structure–activity relationship (SAR) of anthocyanin compounds in a nutraceutical context, we have evaluated the radical scavenging and biological activities of selected purified anthocyanin samples (PASs) from various plant materials. When comparing the anthocyanin sources of our diet, it is obvious that the anthocyanins of vegetables in general are considerably more complex than those of fruits: In vegetables, the proportion of simple anthocyanins without acyl groups and just one or two monosaccharide units is 16%. The corresponding number in the fruits is 74%. Around 70% of anthocyanins in vegetables have one or more aromatic acyl groups, while only 11% of the anthocyanins in the fruits contain an aromatic acyl group [6]. Since there exists a distinct difference between the anthocyanin content in vegetables and fruits of our diet, at least with respect to the aromatic acylation and number of monosaccharide units, we have chosen to compare the activity of PASs from two anthocyanin-rich fruits (mahaleb cherry and blackcurrant) and vegetables (black carrot and “Sun Black” tomato). These samples contain anthocyanins representative of a typical anthocyanin-rich fruit and vegetable diet.

The antioxidative capacities of different PASs, and the anti-inflammatory activity by measuring the expression of endothelial adhesion molecule (VCAM-1 and ICAM-1), in human endothelial cells under inflamed conditions, are reported in relation to the different chemical structures.

## 2. Results and Discussion

### 2.1. Anthocyanin Composition of Selected Fruits and Vegetables

**Figure 1:**
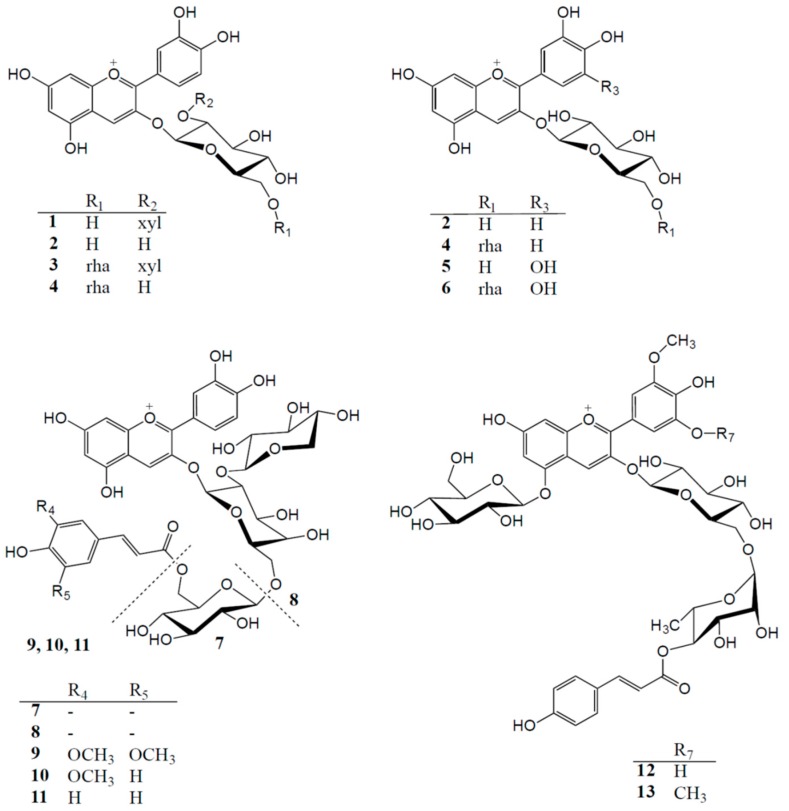
Anthocyanins in purified extracts of mahaleb cherry (**1**–**4**), blackcurrant (**2**, **4**–**6**), black carrot (**7**–**11**) and “Sun Black” tomato (**12**, **13**).

Anthocyanins and other polyphenolic compounds were extracted from (a) mahaleb cherry (*Prunus mahaleb* L.), a marginal fruit crop producing cherry-like dark-purple drupes, rich in non-acylated cyanidin 3-glycosides; (b) blackcurrant (*Ribes nigrum* L.), a well-known berry with high amounts of non-acylated cyanidin and delphinidin 3-glycosides; (c) black carrot (*Daucus carota* L. ssp. *sativus* var. *atrorubens* Alef.), an anthocyanin-rich carrot producing mostly cyanidin 3-glycosides acylated with various cinnamic acid derivatives; and (d) “Sun Black” tomato, a new genotype of tomato synthesising anthocyanins in the peel, based mainly on petunidin and malvidin 3,5-diglycosides acylated with *p*-coumaric acid (Table 1). The relative quantities of the individual anthocyanins in the various PASs are shown in Table 1, while the total anthocyanin contents of the purified samples are shown in Table 2. Accordingly, four groupings of anthocyanins were tested, including two non-acylated anthocyanidin 3-glycosides groupings from fruits (cyanidin-based or delphinidin-based) and two anthocyanidin 3-glycosides groupings acylated with aromatic acyl groups from vegetables. The structural differences of the two acylated anthocyanin groupings reside in the presence or absence of methoxy groups on the anthocyanidin B-ring, in the glycoside type and position (3-glycoside versus 3,5-diglycoside) and the nature of the cinnamic acid involved (Figure 1).

### 2.2. Antioxidative Capacity of Different Anthocyanin Groupings from Selected Fruits and Vegetables

In this study, the radical scavenging activity of purified anthocyanin samples (PASs) from two fruits (mahaleb cherry and blackcurrant) and two vegetables (black carrot and “Sun Black” tomato) have been compared *in vitro* using two of the most commonly used methods (TEAC and ORAC), which account for different mechanisms of action, as recommended by Niki [27].

Mahaleb cherry PAS, which is rich in cyanidin 3-glucoside, cyanidin 3-rutinoside and other cyanidin 3-glycosides, showed a TEAC value of 6.01 ± 0.46 μmol TE/mg PAS and a ORAC value of 15.32 ± 1.73 μmol TE/mg PAS; on molar basis, the values were 3.44 ± 0.31and 8.77 ± 0.63 μmol TE/μmol PAS, respectively (Table 2).

Cyanidin 3-glucoside is the most widely distributed anthocyanin in edible fruits [28]. This anthocyanin has attracted extensive research on its physicochemical behavior, biosynthesis and role in food and health effects [29], and has also been considered the most potent anthocyanin against peroxyl radicals [30]. After administration of ^13^C-labelled cyanidin 3-glucoside in humans, the relative bioavailability was found to be around 12% on the basis of the total elimination of the absorbed ^13^C dose via the urine and breath [31]. This latter work suggested that anthocyanins are more bioavailable than previously perceived, and their metabolites seem to be present in the circulation for 48 h after ingestion. The same group prepared various cyanidin 3-glucoside metabolites, and reported these metabolites to be active at physiological concentration to suppress inflammation in human vascular endothelial cells [32]. However, it has recently been reported that dietary supplementation with mono- or di-glycosylated cyanidins (from blackberry or black raspberry) had no effect on body weight, food intake, body composition and metabolic risk factors (fasting blood glucose and insulin sensitivity) in high-fat diet-fed mice [33].

Blackcurrant PAS contained more delphinidin 3-glycosides (delphinidin 3-glucoside and delphinidin 3-rutinoside) (62%) than cyanidin-glycosides (cyanidin 3-glucoside and cyanidin 3-rutinoside) (35.7%). This sample was compared to mahaleb PAS, which only contains cyanidin 3-glycosides (Table 1); a similar antioxidant capacity was expressed as the TEAC value (6.44 ± 0.51 μmol TE/mg PAS, or 3.89 ± 0.32 μmol TE/μmol PAS) and an increased ORAC value (17.88 ± 1.87 μmol TE/mg PAS, or 11.02 ± 0.62 μmol TE/μmol PAS), particularly when expressed on a molar basis (Table 2). These results indicate the slightly higher antioxidant activity of delphinidin 3-glycosides in comparison with analogous cyanidin 3-glycosides when tested with an ORAC assay. TEAC and ORAC assays are based on different reaction mechanisms: electron transfer for TEAC and hydrogen atom transfer for ORAC. Moreover, results from ORAC reflect more than just a radical scavenging activity, being the only assay that combines both inhibition time and degree of inhibition, ending in a complete reaction [30]. Together these features are responsible for the different values of antioxidant capacity assessed with different assays.

Delphinidin, having three hydroxyl-groups on the B-ring, has been shown to have the highest antioxidant activity among the six most common anthocyanidins [8,12]. Previous studies have revealed that delphinidin-type anthocyanins have shown higher biological activity compared to cyanidin-type anthocyanins. When feeding mice with a 1% blackcurrant diet, weight gain was suppressed and glucose metabolism improved, and the effects were suggested to be exerted by involvement of metabolites generated by enteric bacteria [34]. Moreover, since delphinidin 3-rutinoside (from blackcurrant) was demonstrated not to undergo substantial breakage into degradation products inside the gastrointestinal tract, the biological activities (improvement of insulin sensitivity) following its administration have been associated to this anthocyanin structure by itself [35]. Therefore, the difference in the biological activities of different anthocyanins has been suggested to be related to their structure and their individual metabolism in the gut [33].

The purified anthocyanin samples from black carrot and “Sun Black” tomato peel, containing mainly anthocyanins acylated with cinnamic acid derivatives, showed lower antioxidant activity (except for ORAC, expressed on a molar basis) than the non-acylated anthocyanin groupings from the fruits (Table 2). The black carrot PAS had a TEAC value of 2.53 ± 0.59 μmol TE/mg PAS (2.24 ± 0.28 μmol TE/μmol PAS) and an ORAC value of 12.66 ± 1.86 μmol TE/mg PAS (11.2 ± 0.87 μmolTE/μmol PAS), while “Sun Black” tomato peel PAS had a TEAC value of 1.30 ± 0.13 μmol TE/mg PAS (1.26 ± 0.22 μmol TE/μmol PAS), and an ORAC value of 11.44 ± 1.56 μmol TE/mg PAS (10.68 ± 0.38 μmol TE/μmol PAS). The black carrot sample contains anthocyanins based on the same aglycone (cyanidin) as the mahaleb cherry sample. Although the sugar moieties of the anthocyanins in the two samples are different (Table 1), they are all linked to the cyanidin 3-position. Thus, the main difference between the anthocyanin content of the two samples is the absence of aromatic acylation of all the anthocyanins from mahaleb cherries. Accordingly, the 58% reduction of TEAC activity and 17.4% reduction of ORAC activity of the black carrot PAS relative to the mahaleb cherry PAS indicate the considerable impact of anthocyanin acylation with cinnamic acid derivatives on the antioxidant activity of this type of anthocyanin. The statistically significant difference between the two anthocyanin sources is maintained when the results are expressed on a molar basis. These results are also in accordance with other reports in the field [9,33]. However, when the antioxidant activities of the two major anthocyanins from eggplant, delphinidin 3-rutinoside-5-glucoside and delphinidin 3-(6-(4-(*E*-*p*-coumaroyl)rhamnosyl)glucoside)-5-glucoside (Nasunin) were measured by DPPH assay and linoleic acid radical scavenging activity assays, the non-acylated anthocyanins showed lower activity than the acylated anthocyanin [36]. In some studies, acylated anthocyanins from black carrot have shown less bioavailability compared to non-acylated anthocyanins, probably due to their larger size and different polarity, which prevent their partition into the lipid bilayer or the interaction with bilitranslocase for transport across the gut epithelium [37,38].

The antioxidative capacity of “Sun Black” tomato PAS ranks this source as the lowest (except for ORAC, expressed on a molar basis, due to the high molecular weight of petanin) among the different tested anthocyanin groupings, particularly along the ABTS assay. “Sun Black” tomato is a trademark protected tomato line obtained at Tuscia University (Viterbo, Italy), characterised by deep purple pigmentation in the epicarp due to an increased level of anthocyanins on the peel. Such a line is a product of breeding, and the anthocyanin pigments accumulate in the fruit epidermis and underlying cell layers, particularly on the side most exposed to the sun; instead, the flesh keeps the same red tone as usual [39]. Even though the antioxidative capacity of petanin, the principal anthocyanin found in PAS from the “Sun Black” tomato, resulted in a somewhat lower antioxidative capacity than the other sources, it must be considered that the anthocyanins in this source give increased value to the total antioxidant capacity of this new tomato genotype in comparison with the bioactive compounds normally found in traditional tomatoes.

Acylated anthocyanins (from black carrot, red cabbage, red radish, red and blue potatoes, red corn, etc.) are more suitable than their non-acylated analogues to be applied in food products with pH ranging from acid to neutral and slightly alkaline, due to their higher resistance to colour fading with increased pH [40]. To this list of vegetables providing acylated anthocyanins we can add the “Sun Black” tomato. For several food products these “vegetable anthocyanins” represent attractive alternatives to the addition of synthetic colorants, even if it must be underlined the somewhat less potent antioxidative and biological activities of anthocyanins acylated with cinnamic acid derivatives in comparison with their non-acylated ones (Table 2).

### 2.3. Vascular Anti-Inflammatory Properties of Selected Anthocyanin Groupings in Endothelial Cells

**Figure 2:**
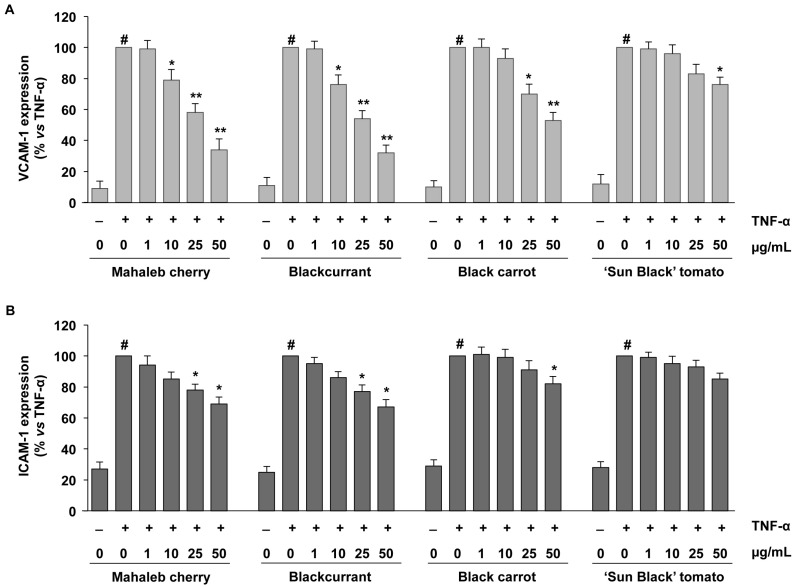
Inhibitory effects of PASs from the mahaleb cherry, blackcurrant, black carrot and “Sun Black” tomato on the expression of endothelial adhesion molecules. Endothelial cells were pre-treated with PASs at different concentrations (1, 10, 25 and 50 μg/mL) or vehicle (control) for 24 h and then stimulated with TNF-α (10 ng/mL) for 16 h. Cell surface expression of VCAM-1 (**A**) and ICAM-1 (**B**) was analysed by cell-surface enzyme immunoassay (EIA). Each experiment was performed in triplicate. Data are expressed as the percentage of TNF-α induced expression (mean ± S.D.). ^#^ *p* < 0.01 vs. control; * *p* < 0.05, ** *p* < 0.01 vs. TNF-α alone.

The vascular protective effects of different anthocyanin groupings from pigment-rich fruits (mahaleb cherry and blackcurrant) and vegetables (black carrot and “Sun Black” tomato) were analysed using a model of vascular inflammation, represented by cultured human microvascular endothelial cells-1 (HMEC-1) challenged with the pro-inflammatory cytokine, tumour necrosis factor-α (TNF-α). In this model, the anti-inflammatory properties of different PASs were evaluated by measuring the TNF-α-stimulated expression of endothelial adhesion molecules, VCAM-1 and ICAM-1. In endothelial cells, treatment with TNF-α induced cell surface expression of VCAM-1 and ICAM-1 (Figure 2). We found that the pre-exposure of endothelial cells with all the various PASs displayed anti-inflammatory properties by inhibiting the TNF-α-induced VCAM-1 and ICAM-1 expression, although their activities were scaled differently (Figure 2). PASs from mahaleb cherry and blackcurrant, containing non-acylated anthocyanins (Table 1), were shown to be the most effective. They significantly reduced the TNF-α stimulated expression of VCAM-1 in a concentration-dependent manner (Figure 2A), with 10 μg/mL as the lowest significant concentration for both PASs. Similarly to VCAM-1, the exposure of endothelial cells to mahaleb cherry and blackcurrant PASs inhibited the TNF-α-induced expression of ICAM-1, although to a lesser degree, with 25 μg/mL as the lowest effective concentration (Figure 2B). Thus, PASs from mahaleb and blackcurrant exhibited similar vascular anti-inflammatory effects, despite their somewhat different anthocyanin composition. Indeed, mahaleb cherry PAS mainly contained cyanidin 3-rutinoside (about 34%) and cyanidin 3-glucoside (about 33%), while blackcurrant PAS contained delphinidin 3-rutinoside (about 56%) and cyanidin 3-rutinoside (about 32%) as major anthocyanins (Table 1). Previous studies have shown vascular anti-inflammatory properties for either cyanidin 3-glycoside or delphinidin 3-glycosides, reporting their ability to decrease the expression of VCAM-1 and ICAM-1 in endothelial cells challenged with several oxidant and inflammatory triggers [32,41,42]. In accordance with the reduced expression of endothelial adhesion molecules, anthocyanins were also able to reduce the adhesion of monocytes to TNF-α-activated endothelial cells at physiologically relevant concentrations, with delphinidin 3-glucoside as the most efficient [43]. The anti-inflammatory effects of anthocyanins have also been shown by their gut metabolites [43], highlighting the beneficial properties of both native and metabolised forms.

Moreover, the present findings confirm our previous study about the ability of mahaleb cherry extract to decrease the stimulated expression of endothelial adhesion molecules [44], and highlight that the mahaleb fraction responsible for anti-inflammatory activity is represented by its anthocyanin-enriched content.

In addition, we analysed the anti-inflammatory properties of samples containing mainly anthocyanins acylated with cinnamic acid derivatives, from both black carrot and “Sun Black” tomato peel. PAS from black carrot efficiently reduced the TNF-α-induced VCAM-1 expression in a concentration-dependent manner, with 25 μg/mL as the lowest effective concentration (Figure 2A). PAS from “Sun Black” tomato inhibited TNF-α-induced VCAM-1 expression significantly only at 50 μg/mL (Figure 2A). In TNF-α stimulated HMEC-1, black carrot PAS was also able to inhibit ICAM-1 expression, but only at the highest concentration (50 μg/mL); “Sun Black” tomato PAS showed a tendency to reduce ICAM-1; however, the inhibitory effect was not statistically significant (Figure 2B).

Overall, all tested PASs were able to decrease (although to a different degree) the stimulated endothelial adhesion molecules’ expression, without affecting the expression of the constitutive endothelial surface antigen E1/1 or the endothelial cell vitality, as determined by cell count and Trypan blue exclusion.

Our findings showed that anthocyanins acylated with cinnamic acid derivatives exhibited a certain degree of anti-inflammatory activity, with lesser efficacy than non-acylated ones. Indeed, when comparing the activity of PASs from mahaleb cherry and black carrot, containing anthocyanins based on the same aglycone (cyanidin) the non-acylated anthocyanins from mahaleb cherry were the most effective in the reduction of the endothelial expression of inflammatory antigens: Mahaleb cherry PAS was effective at concentrations of 10 µg/mL for VCAM-1 and 25 μg/mL for ICAM-1, respectively, while black carrot PAS was effective at 25 µg/mL for VCAM-1 and 50 μg/mL for ICAM-1, respectively.

The differences in biological properties observed for the non-acylated and acylated anthocyanins were related to the antioxidant activity of their respective PASs, with the non-acylated anthocyanins in the mahaleb cherry and blackcurrant samples having the most effective activity, followed by the anthocyanins acylated with cinnamic acid derivatives in the black carrot and lastly “Sun Black” tomato samples. Notably, in this study we analysed the biological activities of different PASs by referring to the dry weight of each extract (µg of PAS in mL of medium, Figure 2). Comparing different PASs at the same concentration expressed as µg/mL (Table 3), the anthocyanins with acylation had less molar concentration than the non-acylated anthocyanins because of higher molecular weight, which might partially explain their lower efficacy. In any case, our results regarding the endothelial anti-inflammatory effect of anthocyanins with aromatic acylation from vegetables were supported by the in vivo study showing that purple sweet potato anthocyanins suppressed the development of atherosclerotic lesions and enhancements of oxidative stress and soluble VCAM-1 in an animal model of atherosclerosis [19].

Overall, our findings show that anthocyanins may play a positive role in the context of cardiovascular health due to their anti-inflammatory and anti-atherosclerotic effects. However, comparative analyses suggested that the anthocyanin structure affected the protective action, with non-acylated anthocyanins having a greater inhibitory effect on TNF-α-induced expression of VCAM-1 and ICAM-1 in endothelial cells than anthocyanins acylated with cinnamic acid derivatives. These findings could be useful for food valorization and the development of anthocyanin-rich functional foods.

## 3. Materials and Methods

### 3.1. Standards and Chemical Reagents

Reagents were purchased from various suppliers as follows: authentic standards of kuromanin chloride (cyanidin 3-*O*-glucoside chloride), keracyanin chloride (cyanidin 3-*O*-rutinoside chloride), myrtillin chloride (delphinidin 3-*O*-glucoside chloride), delphinidin 3-*O*-rutinoside chloride, *p*-coumaric acid, chlorogenic acid (3-caffeoylquinic acid), rutin (quercetin 3-*O*-rutinoside), and isoquercitrin (quercetin 3-*O*-glucoside) (Extrasynthèse, Genay, France); Trolox ((*S*)-(−)-6-hydroxy-2,5,7,8-tetramethylchroman-2-carboxylic acid), FL (fluorescein disodium), ABTS (2,2′-azino-bis (3-ethylbenzothiazoline-6-sulfonic acid)), AAPH (2,2′-azobis (2-methyl-propionamidine dihydrochloride)), as well as acetonitrile (HPLC grade), ethanol, methanol, formic acid (Sigma-Aldrich, St. Louis, MO, USA). In all experiments Milli-Q (Merck Millipore, Darmstadt, Germany) water was used.

Petunidin 3-(6-(4-(*E*-*p*-coumaroyl)rhamnosyl)glucoside)-5-glucoside (Petanin) (**12**) and malvidin 3-(6-(4-(*E*-*p*-coumaroyl)rhamnosyl)glucoside)-5-glucoside (Negretein) (**13**) were isolated from blue potatoes (*Solanum tuberosum* cv. Congo and *S. tuberosum* cv. Vitelotte noire, respectively): Diced and frozen potatoes were extracted three times with acidified methanol (0.5% TFA), and the combined filtered extract was purified using partition against ethyl acetate followed by Amberlite XAD-7 column chromatography. Petanin and Negretein were isolated from their respective purified extracts using Sephadex LH-20 column chromatography and preparative HPLC. Their structures were elucidated by 1D and 2D NMR and high-resolution mass spectrometry (MS).

### 3.2. Plant Material, Preparation of Purified Anthocyanin Samples (PASs) and Anthocyanin Identification

**Figure 3:**
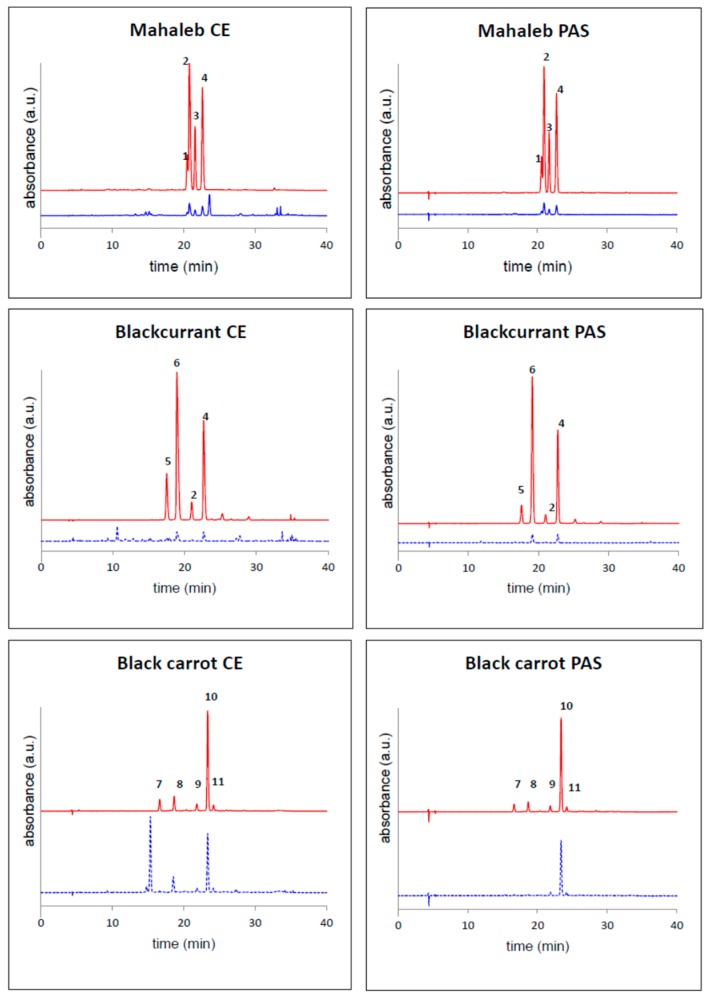
HPLC chromatograms of crude extracts (CE) and purified anthocyanin samples (PASs) of mahaleb cherry, blackcurrant and black carrot, detected at λ = 520 nm (red line) and 280 nm (blue line). See Table 1 for identities.

All fruits and vegetables were harvested at their ripened stage. Extractions from respectively mahaleb cherry (*Prunus mahaleb* L.), blackcurrant (*Ribes nigrum* L.), black carrot (*Daucus carota* L. ssp. *sativus* var. *atrorubens* Alef.) and “Sun Black” tomato (*Solanum lycopersicum* L.) peel were done from 500 mg dry weight (DW) plant material, macerated with 50 mL extraction solvent (35% methanol + 35% ethanol + 28% water + 2% formic acid), at 4 °C, overnight. After centrifugation of the slurry (10 min at 2000 *g*), the supernatant was collected. A further 50 mL of extraction solvent was added to the pellet, and the extraction was repeated on a rotary shaker for one hour. Pooled supernatants were evaporated *in vacuo* at 32 °C using a R-205 Büchi rotavapor (Büchi Labortechnik AG, Flawil, Switzerland) and re-suspended in acidified water (0.5% formic acid). This crude extract (CE) was purified by solid-phase extraction, using C-18 cartridge solid phase extraction (SPE) (STRATA C-18E, Phenomenex, Torrance, CA, USA). Firstly, washing with acidified water (0.5% formic acid) removed water-soluble compounds, and secondly, a mixture of water:ethyl acetate:methanol (35:45:20) removed non-anthocyanin polyphenols, and finally methanol:water:formic acid (40:59.5:0.5) eluted the purified anthocyanins. The respective purified anthocyanin samples (PASs) were concentrated *in vacuo* at 34 °C, and characterised by HPLC (Figure 3). The respective CEs were also filtered through a 0.45 μm CA syringe filter (Filtres Fioroni, Ingré, France), portioned and stored at −20 °C before HPLC analysis (Figure 3).

The individual anthocyanins in the respective PASs (Table 1 and Figure 1) were identified by a combination of co-chromatography (diode-array detection (DAD) during HPLC) with authentic standards and comparison with the literature: mahaleb cherry [45], blackcurrant [46] and black carrot [47]. The two main anthocyanins in “Sun Black” tomato PAS were identified to be petanin (**12**) [48] and negretein (**13**) [49] by using DAD-HPLC and high-resolution LC-MS in comparison with authentic standards.

### 3.3. Quantification of Anthocyanin Content

The relative quantities of the individual anthocyanins in the PASs were established by integration of HPLC peaks detected at λ = 520 ± 20 nm. The total anthocyanin content of the CEs as well as PASs were quantified by HPLC, using a standard curve based on the main anthocyanin in the sample (if commercially available) or, in the case of black carrot and “Sun Black” tomato PASs, based on the cyanidin 3-glucoside standard. The amounts of each anthocyanin calculated as an external standard equivalent were then multiplied by a molecular weight correction factor [50].

### 3.4. Antioxidant Activity Assays

The anthocyanin content of the PASs was normalised to 60 μg/mL and tested at suitable dilutions. The antioxidant capacity of the PASs was measured using the ABTS (TEAC) and the ORAC assays as described by Gerardi et al. (2015) [51], and expressed as a function of the Trolox reference standard (μmol Trolox equivalents (TE)/g of DW). A rapid microplate methodology, using a microplate reader (Infinite M-200, Tecan Group Ltd., Männedorf, Switzerland) and 96-well plates (Costar, 96-well clear round bottom plate, Corning) was applied. All experiments were performed in triplicate, and at least two independent assays were performed for each sample. The activity was also calculated on a molar basis using a weighted average of the molar weights of the pigments in each PAS and the identified relative quantities (assuming the un-identified portion is similar to the average).

### 3.5. Cell Culture and Treatments

The human microvascular endothelial cell line (HMEC-1), obtained from Dr. Thomas J. Lawley, was cultured as previously described [52]. Before treatment, confluent cells were shifted to media containing 4% foetal bovine serum, and incubated in the absence (vehicle) or presence of PASs at different concentrations (final concentrations of 1, 10, 25 and 50 μg/mL cell culture medium) for 24 h, and then stimulated with TNF-α (10 ng/mL) for additional 16 h. The effects of vehicle control or different PASs on cell viability were evaluated through a variety of techniques, including cell count and Trypan blue exclusion. In preliminary experiments aimed at evaluating phytochemical toxicity, treatment of HMEC-1 with up to 50 μg/mL PAS for 24 h did not produce any sign of toxicity.

### 3.6. Detection of Endothelial Cell Molecules

Endothelial surface expression of VCAM-1 and ICAM-1 was assayed by employing a cell surface enzyme immunoassay (EIA), using primary mouse anti-human monoclonal antibodies against VCAM-1 (Millipore) and ICAM-1 (HU5/3), or the monoclonal antibody against the non-cytokine-inducible and constitutive endothelial cell antigen E1/1, as previously described [53].

### 3.7. Statistical Analysis

Values were expressed as mean ± SD for the indicated number of experiments. Differences between two groups were settled by unpaired Student’s *t* test. One-way analysis of variance (ANOVA) followed by the Fisher’s least significant difference (LSD) post hoc test was used to determine significant differences among groups.

## 4. Conclusions

In the present study we demonstrated that purified anthocyanin samples from four plant sources (mahaleb cherry, blackcurrant, black carrot and “Sun Black” tomato) containing structurally different anthocyanins, were biologically active. They all exhibited antioxidant capacity, which was highest for samples containing non-acylated anthocyanins versus samples containing anthocyanins with aromatic acylation, even though this order was reversed when ORAC assay values were expressed on a molar basis. These purified anthocyanin samples were also able to reduce the expression of endothelial inflammatory antigens, suggesting their potential beneficial effect in cardiovascular protection. When compared, the vascular anti-inflammatory capacity of non-acylated anthocyanins was higher than the similar capacity of anthocyanins acylated with cinnamic acid derivatives in accordance with their corresponding antioxidant activity. Future studies might better clarify the underlying mechanisms of structurally different anthocyanins related to vascular health.

## Figures and Tables

**Table 1:** Relative anthocyanin proportions in purified extracts of mahaleb cherry, blackcurrant, black carrot and “Sun Black” tomato.

Source	%
**Mahaleb Cherry**	
Cyanidin 3-(6-(rhamnosyl)glucoside) (4)	34.3
Cyanidin 3-glucoside (2)	33.4
Cyanidin 3-(6-(rhamnosyl)-2-(xylosyl)glucoside) (3)	21.3
Cyanidin 3-(2-(xylosyl)glucoside) (1)	10.9
**Blackcurrant**	
Delphinidin 3-(6-(rhamnosyl)glucoside) (6)	56.1
Cyanidin 3-(6-(rhamnosyl)glucoside) (4)	32.4
Delphinidin 3-glucoside (5)	5.9
Cyanidin 3-glucoside (2)	3.3
**Black Carrot**	
Cyanidin 3-(6-(6-(feruloyl)glucosyl)-2-(xylosyl)galactoside) (10)	77.1
Cyanidin 3-(6-(6-(sinapoyl)glucosyl)-2-(xylosyl)galactoside) (9)	9.9
Cyanidin 3-(2-(xylosyl)galactoside) (8)	4.9
Cyanidin 3-(6-(glucosyl)-2-(xylosyl)galactoside) (7)	4.8
Cyanidin 3-(6-(6-(*p*-coumaroyl)glucosyl)-2-(xylosyl)galactoside) (11)	3.1
**“Sun Black” Tomato**	
Petunidin 3-(6-(4-(*E*-*p*-coumaroyl)rhamnosyl)glucoside)-5-glucoside (petanin) (12)	56.6
Malvidin 3-(6-(4-(*E*-*p*-coumaroyl)rhamnosyl)glucoside)-5-glucoside (13)	21.4
Unknown	22.0

**Table 2:** Anthocyanin content (TA) in purified anthocyanin samples (PASs) of mahaleb cherry, blackcurrant, black carrot and “Sun Black” tomato (as dry weight, DW) and their antioxidant activity measured in TEAC and ORAC assays.

Source	TA	TEAC	TEAC	ORAC	ORAC
	mg AntE /g DW	μmol TE/mg PAS	μmol TE/μmol PAS	μmol TE/mg PAS	μmol TE/μmol PAS
**Mahaleb cherry**	38.5 ± 1.50 ^a^	6.01 ± 0.46 ^a^	3.44 ± 0.31 ^a^	15.32 ± 1.73 ^a b^	8.77 ± 0.63 ^b^
**Blackcurrant**	32.2 ± 2.33 ^b^	6.44 ± 0.51 ^a^	3.89 ± 0.32 ^a^	17.88 ± 1.87 ^a^	11.02 ± 0.62 ^a^
**Black carrot**	12.1 ± 0.48 ^c^	2.53 ± 0.59 ^b^	2.24 ± 0.28 ^b^	12.66 ± 1.86 ^b^	11.20 ± 0.87 ^a^
**“Sun Black” tomato**	4.9 ± 0.26 ^d^	1.30 ± 0.13 ^c^	1.26 ± 0.22 ^c^	11.44 ± 1.56 ^b^	10.68 ± 0.38 ^a^
**Significance**	***	***	***	**	**

AntE means anthocyanin equivalent; *** and ** significant at *p* ≤ 0.001 and 0.01, respectively. For each parameter, the same letters in the same column indicate that mean values are not significantly different (*p* = 0.05).

**Table 3:** Molar concentration (μmol/L) of purified anthocyanin samples (PAS) used in cell culture experiments.

PAS	μmol/L			
Mahaleb cherry	1.7	17.5	43.6	87.3
Blackcurrant	1.6	16.2	40.5	81.1
Black carrot	1.1	11.3	28.2	56.5
“Sun Black” tomato	1.1	10.7	26.8	53.5
μg/mL	1	10	25	50

## Abbreviations

PAS	Purified Anthocyanin Sample
TEAC	Trolox Equivalent Antioxidant Capacity
ORAC	Oxygen Radical Absorbance Capacity
VCAM-1	Vascular Cell Adhesion Molecule-1
ICAM-1	Intercellular Adhesion Molecule-1
HMEC-1	Human Microvascular Endothelial Cell-1
TNF-α	Tumour Necrosis Factor-α
EIA	Cell-Surface Enzyme Immunoassay
